# Point of care coagulometry in prehospital emergency care: an observational study

**DOI:** 10.1186/s13049-015-0139-6

**Published:** 2015-08-12

**Authors:** Christopher Beynon, Angelina G. Erk, Anna Potzy, Stefan Mohr, Erik Popp

**Affiliations:** Department of Neurosurgery, Heidelberg University Hospital, Im Neuenheimer Feld 400, 69120 Heidelberg, Germany; Department of Anaesthesiology, Heidelberg University Hospital, Im Neuenheimer Feld 110, 69120 Heidelberg, Germany

## Abstract

**Background:**

Haemostatic impairment can have a crucial impact on the outcome of emergency patients, especially in cases of concomitant antithrombotic drug treatment. In this prospective observational study we used a point of care (POC) coagulometer in a prehospital physician-based emergency medical system in order to test its validity and potential value in the treatment of emergency patients.

**Methods:**

During a study period of 12 months, patients could be included if venous access was mandatory for further treatment. The POC device CoaguChek® was used to assess international normalized ratio (INR) after ambulance arrival at the scene. Results were compared with in-hospital central laboratory assessment of INR. The gain of time was analysed as well as the potential value of POC testing through a questionnaire completed by the responsible prehospital emergency physician.

**Results:**

A total of 103 patients were included in this study. POC INR results were highly correlated with results of conventional assessment of INR (Bland-Altman-bias: 0.014). Using a cutoff value of INR >1.3, the device’s sensitivity to detect coagulopathy was 100 % with a specificity of 98.7 %. The median gain of time was 69 min. Treating emergency physicians considered the value of prehospital POC INR testing ‘high’ in 9 % and ‘medium’ in 21 % of all patients. In patients with tracer diagnosis ‘neurology’, the value of prehospital INR assessment was considered ‘high’ or ‘medium’ (63 %) significantly more often than in patients with non-neurological tracer diagnoses (24 %).

**Conclusions:**

Assessment of INR through a POC coagulometer is feasible in prehospital emergency care and provides valuable information on haemostatic parameters in patients. Questionnaire results suggest that POC INR testing may present a valuable technique in selected patients. Whether this information translates into an improved management of respective patients has to be evaluated in further studies.

## Introduction

Impaired haemostasis can have a crucial impact on the outcome of emergency patients. Trauma-associated bleeding is considered a major risk factor for increased mortality and even small blood clots can have devastating effects if they are localized within the central nervous system. Furthermore, antithrombotic agents are increasingly prescribed as the population ages and anticoagulant-associated coagulopathy may have detrimental effects in patients with acute haemorrhage [1].

Point of care (POC) devices have been developed for patient self-monitoring of anticoagulation therapy with vitamin K antagonists (VKA). Assessment of prothrombin time international normalised ratio (INR) is achieved within one minute through analysis of a drop of blood and results have been highly correlated with results of conventional assessment of INR [2, 3]. Several reports have demonstrated the value of an in-hospital use of POC devices for rapid assessment of INR. In emergency settings, their use has reduced the ‘door to needle’ time for thrombolysis in acute ischemic stroke [4] or urgent neurosurgical intervention in anticoagulated patients [5]. Theoretically, prehospital assessment of INR may further optimize the emergency management of respective patients and may also facilitate prehospital administration of pro-haemostatic substances.

The primary aim of this prospective observational study was to test the potential value of prehospital INR assessment through emergency medical service (EMS). For this purpose we used a POC device in a physician-based EMS ambulance and analysed the gain of time and accuracy of obtained results compared with conventional central laboratory (CL) assessment of INR. For each patient included in this study, the EMS physician completed a questionnaire regarding the potential value of INR assessment in the specific case.

## Methods

### Study design and data collection

The study was approved by the local Ethics Committee (S-085/2013). All patients (>18 years) treated by a physician-based prehospital EMS were eligible for inclusion if a venopuncture was performed during treatment in order to establish intravenous access. Patients could be included if a physician briefed in the use of the device was present and logistical circumstances allowed study inclusion. Written consent to study participation was obtained either prior to study participation or retrospectively after recovery of consciousness. After insertion of an intravenous line (Braunüle®, BBraun, Melsungen, Germany), a drop of blood was collected from the needle and was applied on the test strip of the POC device for assessment of INR. The point of time and result of INR assessment were documented. The results were not used to guide further patient treatment. After patient admission to the hospital, the EMS emergency physician completed a questionnaire regarding the potential value of prehospital INR assessment in the treatment of the specific patient. INR values of laboratory examination and time points of availability were provided by respective hospitals. The cutoff value for ‘coagulopathic’ was defined at INR > 1.3.

### Point of care device for assessment of prothrombin time

The POC device CoaguChek XS® (Roche Diagnostics, Mannheim, Germany) was used in this study. It is a small (128×78×28 mm), battery-operated and portable device which can be easily used through a screen-based operating system. It uses the amperometric detection of thrombin in a 10 μL sample of whole blood with a disposable test strip and measures INR from 0.8 to 8.0. INR values outside this range may produce test errors indicated by ‘error’. Blood has to be applied on the test strip within 2 min after venopuncture. Thereafter, initiated coagulation processes may produce errors. Environmental temperature has to be within 5 °C to 35 °C because of enzymatic reactions incorporated on the test strip during assessment of INR. In those cases, ‘error’ is displayed and INR assessment is not initiated.

### Questionnaire

The questionnaire included three questions regarding the potential value of INR assessment in the specific case and was completed by the EMS physician directly after hospital admission of the patient: Question 1: ‘How do you rate the overall value of prehospital assessment of INR in the treatment of this patient (1: no, 2: low, 3: medium, 4: high)’? Question 2: ‘Would you have suspected coagulopathy in this patient after obtaining medical history and clinical examination (yes/no)’? Question 3: ‘Would you have considered prehospital administration of prothrombin complex concentrate in this patient (yes/no)’?

### Statistical analysis

Descriptive statistical analyses were used for numerical values. POC INR and CL INR values were compared with the Spearman’s rank correlation test. To test the agreement between both methods, we carried out a Bland-Altman analysis^9^. For comparison of questionnaire results (Question 1), the Fisher exact test was carried out between results of patients with a neurological tracer diagnosis and patients with non-neurological tracer diagnoses results (‘high’/’medium’ value vs. ‘low’/’no’ value). A p-value <0.05 was considered statistically significant. All data were analysed with the statistical software GraphPad Prism 5 (GraphPad Software, La Jolla, USA).

## Results

A total of 103 patients (male: 53, female: 50) were included in this study. Patient characteristics are summarized in Table 1. Tracer diagnosis categories for ambulance calls were cardiovascular (*n* = 39), trauma (*n* = 19), neurology (*n* = 16), respiratory (*n* = 9) and other (*n* = 20).

**Table 1:** Patient characteristics and results of point-of-care (POC) testing as well as agreement with central laboratory assessment of international normalized ratio (INR) and time gained through the use of the device

Tracer diagnosis	Age	Age	POC INR	POC INR	CL INR	CL INR	Bland-Altman	Time gain	Time gain
	Median	Range	Median (IQR)	Range	Median (IQR)	Range	bias	Median (IQR)	Range
Cardiovascular	77	35 – 88	1.10 (1.0 – 1.2)	0.8 – 3.3	1.04 (0.97 – 1.13)	0.94 – 3.13	0.019	67 (56 – 85)	41 – 318
(*n* = 39)									
Trauma	62	29 – 93	1.00 (1.0 – 1.2)	0.9 – 2.2	1.00 (0.98 – 1.10)	0.90 – 1.84	0.053	63 (39 – 161)	33 – 336
(*n* = 19)									
Neurology	64	18 – 89	1.10 (1.0 – 1.5)	0.9 – 3.9	1.07 (0.99 – 1.34)	0.88 – 4.94	0.092	67 (51 – 78)	41 – 143
(*n* = 16)									
Respiratory	72	28 – 89	1.10 (1.1 – 1.5)	1.0 – 2.7	1.00 (0.95 – 1.03)	0.93 – 1.04	0.100	78 (53 – 155)	37 – 263
(*n* = 9)									
Other	59	20 – 81	1.00 (1.0 – 1.1)	0.9 – 3.0	1.00 (0.96 – 1.05)	0.91 – 2.89	0.038	75 (57 – 98)	34 – 211
(*n* = 20)									
All	69	18–93	1.10 (1.0 – 1.2)	0.8 – 3.9	1.03 (0.98 – 1.13)	0.88 – 4.94	0.014	69 (54 – 99)	33 – 336
(*n* = 103)									

**Fig. 1:**
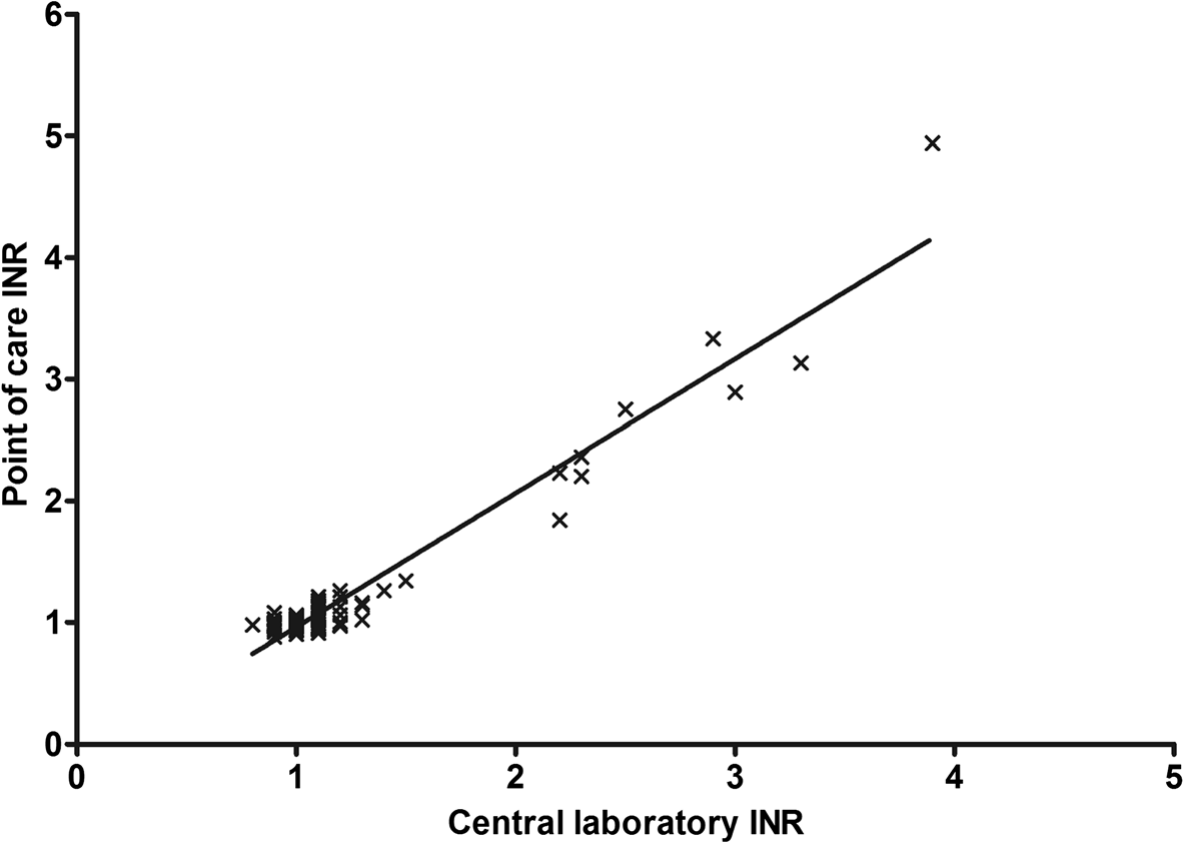
Scatter graph of Spearman’s correlation. The r value of international normalized ratio (INR) results of both test methods was 0.68

**Fig. 2:**
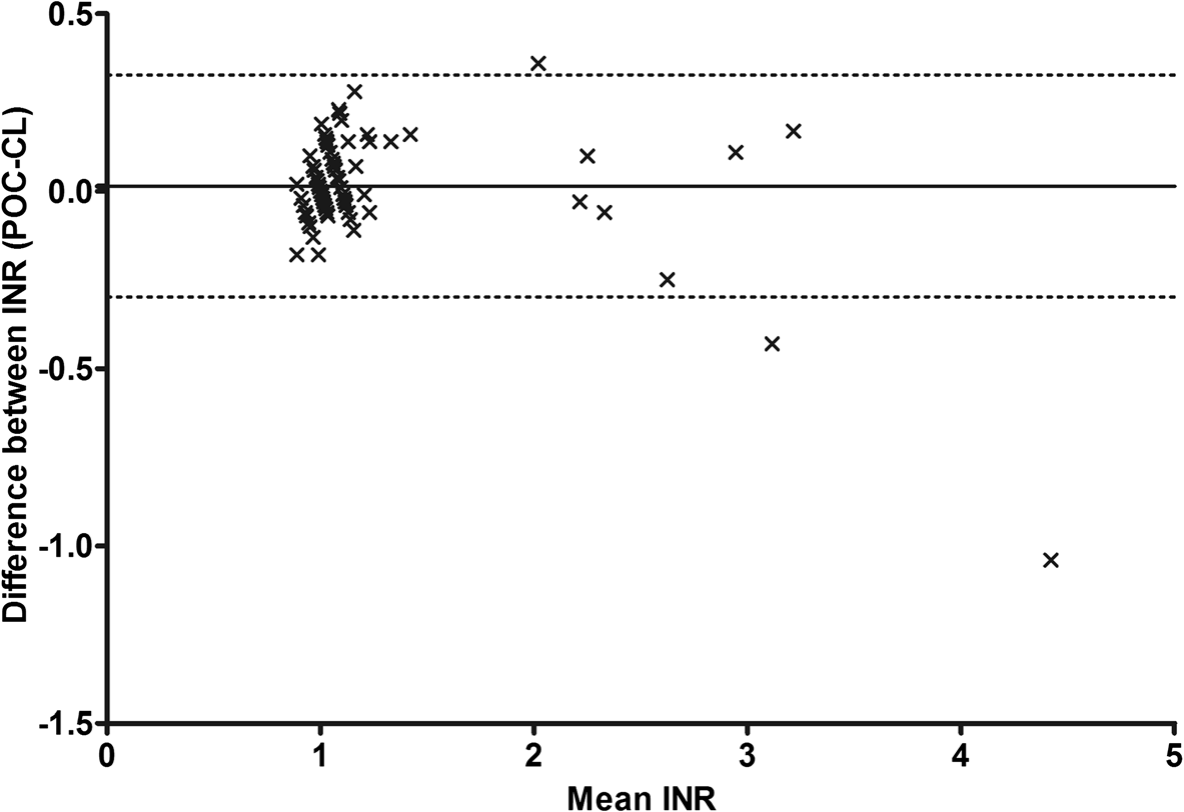
The Bland-Altman-Plot demonstrates the agreement between point-of-care (POC) and central laboratory (CL) assessment of international normalized ratio (INR). The mean bias was 0.014 (continuous line) with 95 % limits of agreement of −0.30 to +0.33 (dotted lines)

Technical difficulties occurred when temperature levels were lower than 5 °C or above 35 °C. In these cases, an error message was displayed. ‘Error message 5’ was displayed in cases of insufficient blood amount indicating errors in applying blood. The median INR obtained through POC coagulometry in patients was 1.10 [IQR: 1.0-1.2]. CL INR assessment upon hospital arrival was carried out in 89 patients (median INR: 1.03 [IQR: 0.98-1.13]). The correlation between POC INR and CL INR was 0.68 (*p* < 0.0001, Fig. 1). Bland Altman analysis revealed a bias of 0.014 with 95 % limits of agreement between −0.30 and +0.33 (Fig. 2). The variability of POC INR increased with higher INR results. POC INR result was 1.4 in one patient while CL INR was 1.26 and therefore, this patient was falsely classified as ‘coagulopathic’ by POC coagulometry. All patients with CL INR > 1.3 were correctly identified as ‘coagulopathic’ by POC coagulometry. POC testing had a sensitivity of 100 % and a specificity of 98.7 % for detecting coagulopathy (Table 2). All patients with coagulopathy had been treated with VKA. The median time gained through POC testing compared with CL assessment of INR was 69 min, ranging from 33 to 336 min).

**Table 2:** Point-of-care (POC) testing international normalized ratio (INR) had a specificity of 100 % and a sensitivity of 98.7 % in detecting coagulopathy, defined as INR >1.3 through central laboratory (CL) assessment

	CL coagulopathic	CL non-coagulopathic	Total
	(INR >1.3)	(INR ≤1.3)	
POC coagulopathic	10	1	11
(INR >1.3)			
POC non-coagulopathic	0	78	78
(INR ≤1.3)			
Total	10	79	

Questionnaire results demonstrated that POC INR results were considered of ‘high’ or ‘medium’ value in the treatment of 31 patients (30 %) (Table 3). In patients with neurological tracer diagnoses, POC INR results were considered significantly more often ‘high’ or ‘medium’ than in patients with a non-neurological tracer diagnosis (63 % vs. 24 %) (*p* < 0.05). Prehospital administration of prothrombin complex concentrate (PCC) would have been considered in a 78-year-old male patient with active upper gastrointestinal bleeding. In this patient, POC testing revealed an INR of 3.0 and CL INR result was 2.89 after admission to the hospital.

**Table 3:** Questionnaire results regarding the value of prehospital assessment of international normalized ratio (INR)

Tracer diagnosis	Questionnaire (1)				Questionnaire (2)	Questionnaire (3)
	Value of INR assessment in treatment of patient				Disagreement of clinical vs. POC assessment of coagulopathy	Consideration of prehospital administration of prothrombin complex concentrate
	No	Low	Medium	High		
Cardiovascular (n=39)	16 (41 %)	16 (41 %)	5 (13 %)	2 (5 %)	1 (3 %)	0
Trauma (n=19)	4 (21 %)	8 (42 %)	5 (26 %)	2 (11 %)	1 (6 %)	0
Neurology (n=16)	3 (19 %)	3 (19 %)	7 (44 %)	3 (19 %)	3 (19 %)	0
Respiratory (n=9)	3 (33 %)	3 (33 %)	2 (22 %)	1 (11 %)	2 (22 %)	0
Other (n=20)	13 (65 %)	3 (15 %)	3 (15 %)	1 (5 %)	0 (0 %)	1
All (n=103)	39 (38 %)	33 (32 %)	22 (21 %)	9 (9 %)	7 (7 %)	1

## Discussion

In prehospital emergency care, impaired haemostasis can have a major impact on patient outcomes. Guidelines for the management of bleeding in major trauma patients recommend ‘monitoring and measures to support coagulation initiated as soon as possible’ [6]. Other scenarios that involve management of haemorrhage and commonly occur in prehospital emergency care include bleeding from the upper gastrointestinal or nose and throat tract. Results of several studies have suggested beneficial effects of an in-hospital use of POC devices for assessment of coagulation. The emergency management of anticoagulated patients is significantly hastened in cases of ischaemic stroke [4], intracerebral haemorrhage [7] and conditions requiring urgent neurosurgical intervention [5]. In those studies, test results were highly correlated with results of conventional INR assessment, confirming previous findings of studies on the validity of test results in the outpatient setting [2, 3].

Only very few reports on the assessment of haemostatic parameters in prehospital emergency care currently exist. We have previously reported a case of preshospital identification of excessive anticoagulation through POC coagulometry in a patient with active bleeding [8]. Lendrum et al. reported the case of CoaguChek®-guided INR assessment by a helicopter-based EMS in an anticoagulated head-injured patient who received prothrombin complex concentrate prior to hospital admission in order to normalize haemostasis [9]. A further study on the prehospital thrombolysis of patients with ischaemic stroke in a CT scanner-equipped ambulance also included POC assessment of INR [10].

The findings of our study demonstrate that prehospital assessment of INR is feasible with the use of a POC coagulometer. Technical difficulties mainly occurred when manufacturer’s instructions were not followed. In cases of outdoor temperature of below 5 °C or higher than 35 °C, INR assessment was not possible as well as when blood was applied later than two minutes after venopuncture. An error message was displayed in those cases and INR assessment was not initiated. Importantly, if INR assessment was initiated by the device and results were obtained, they were highly correlated with conventional laboratory assessment of INR. However, limited numbers of patients with high INR were included in the sample and Bland-Altman analysis suggested greater variability in correlation at high INR. The mean INR deviation was 0.014 with 95 % agreement of −0.30 to +0.33 and those results are considered acceptable in clinical practice. The majority of patients had no signs of coagulopathy with normal INR measured by POC and CL, but all patients with coagulopathy (INR >1.3) had been identified by prehospital coagulometry.

Our findings also demonstrate that POC coagulometry is associated with a major gain of time regarding the assessment of haemostasis in emergency patients. In this study, the median time gained through the use of the POC INR device was 69 min and this gained time can provide substantial benefits in cases of emergency scenarios such as intracranial haemorrhage. Early assessment of INR in selected patients may also aid in transfer decisions as accuracy of prehospital triage positively affects survival and resource utilization [11]. This especially applies to rural areas with limited availability of Level I emergency centres. Prehospital assessment of INR may also hasten the further patient management with regard to haemostatic measures after hospital admission. Furthermore, the prehospital administration of PCC based on POC coagulometry findings could have distinct advantages. PCC formulas do not have to be thawed and enable a rapid reversal of anticoagulation. In our patient collective, prehospital administration of PCC would have been considered by the EMS physician in one patient who suffered from active upper GI bleeding and an INR of 3.0 as revealed by POC coagulometry. The CRASH-2 trial indicated that prehospital administration of the antifibrinolytic drug tranexamic acid reduces mortality in bleeding trauma victims [12] and those findings underscore the potential of haemostatic therapy in prehospital emergency care. POC devices for assessment of haemostasis may play a key role in optimising prehospital coagulation therapy [13].

There are several limitations of our study. Although patients were enrolled prospectively, they were not recruited consecutively. Statistical analyses regarding the validity of test results were carried out, but no patient had significant coagulopathy of an origin other than VKA-associated (eg, traumatic coagulopathy). Therefore, it is not possible to draw conclusions on the system’s capability of detecting other coagulopathies. Assessment of INR reflects only a proportion of the complex pathophysiological cascades of haemostasis. Although INR is suitable to assess the degree of anticoagulation in patients treated with VKA, it is not able to reflect various other sources of coagulopathy. Available studies on the identification of traumatic coagulopathy by POC coagulation testing have reported conflicting results regarding this issue. Cotte et al. have reported the use of CoaguChek® in a military setting and included 40 patients with war trauma [14]. In total, 69 measurements were compared with laboratory assessment of prothrombin time. The Bland-Altman showed a difference of 5.8 % (95 % confidence interval −14.9 % to +26.6 %). A prospective study by Mitra and colleagues included 72 trauma patients of whom 38 were considered coagulopathic (INR > 1.5) [15]. Results of the CoaguChek XS® were obtained upon hospital arrival of patients and compared with standard laboratory assessment of INR. The Bland-Altman plot demonstrated a mean difference of −0.1 with limits of agreement at −1.6 and +1.3. The POC INR had a specificity of 86.1 % and a sensitivity of 63.9 %. The authors concluded that POC INR measurements during trauma reception cannot be used to identify patients with traumatic coagulopathy. Several factors such as hemodilution and disseminated intravascular coagulation have been discussed as reasons for discrepancy to the results of studies on the accuracy of POC INR testing in anticoagulated patients. As recently reviewed by Schött, viscoelastic and platelet function monitoring may also represent suitable techniques for prehospital POC coagulation testing [16]. A further limitation of our study is that CL INR assessment after hospital arrival of the patient was carried out with a significant delay compared with POC INR testing. As haemostasis is a dynamic process, haemostatic parameters could change even within short periods of time and this especially applies to trauma patients with active bleeding.

Despite these limitations, we present the first study on prehospital POC coagulometry in emergency patients and our findings support the potential of POC coagulation testing to optimise treatment modalities of selected patients. The questionnaire results demonstrate that the value of prehospital INR assessment was considered highest in patients with the tracer diagnosis category ‘neurology’. As patients with anticoagulant therapy are particularly prone to ischemic and hemorrhagic strokes, this supports the hypothesis that prehospital INR assessment may enhance the prehospital emergency care of selected patients treated with oral anticoagulants. While current techniques allow assessment of anticoagulation degree, further research is needed to define its value in the treatment of patients with coagulopathy of other origin. Prehospital administration of PCC based on coagulometry findings may improve outcome of anticoagulated patients with severe haemorrhage or intracranial bleeding but this hypothesis has to be proven in future research.

## Conclusion

Prehospital coagulometry by EMS is feasible and enables rapid INR assessment prior to hospital admission of patients. The considerable time gained may have beneficial effects on the treatment of emergency patients. Although INR test results have been validated for assessment of anticoagulation in VKA-treated patients, the accuracy in patients with coagulopathy of other origin is unclear. Further research is needed to characterise the potential and limitations of POC coagulation testing devices in prehospital emergency care.
